# Chronic vertebrobasilar insufficiency in subclavian steal syndrome

**DOI:** 10.1002/ccr3.3891

**Published:** 2021-02-04

**Authors:** Stephanie Phillips, Anza B. Memon

**Affiliations:** ^1^ Department of Neurology Henry Ford Hospital Detroit Detroit MI USA; ^2^ School of Medicine Wayne State University Detroit MI USA

**Keywords:** all neurology, chronic vertebrobasilar insufficiency, chronic vertigo, subclavian steal

## Abstract

Subclavian steal syndrome is a vascular disorder that consists of significant blood supply restriction with resultant insufficiency of the vertebrobasilar artery and the subclavian artery causing symptomatic insufficiency to the brain and upper extremity. It is important to recognize this condition in patients with subacute to chronic posterior circulation vascular insufficiency as early diagnosis and treatment can have good clinical outcomes (J Clin Neurosci. 2010;**17**:1339). CT angiogram of the head and neck should be considered in patients with chronic vertebrobasilar insufficiency to evaluate subclavian steal syndrome.

## Graphical abstract

Subclavian steal syndrome is a vascular disorder that consists of significant blood supply restriction with resultant insufficiency of the vertebrobasilar artery and the subclavian artery causing symptomatic insufficiency to the brain and upper extremity. It is important to recognize this condition in patients with subacute to chronic posterior circulation vascular insufficiency as early diagnosis and treatment can have good clinical outcomes. CT angiogram of the head and neck should be considered in patients with chronic vertebrobasilar insufficiency to evaluate subclavian steal syndrome.

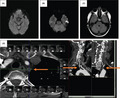

A 60‐year‐old woman with a long‐standing history of seizures (for 20 years) stable on phenytoin (400 mg qhs) presented with subacute progressive vertigo, binocular diplopia, and gait ataxia. Her vital signs were normal at presentation. On examination, the patient was noted to have horizontal gaze nystagmus, mild gait ataxia, and upper and lower extremity ataxia worse on the right side. Computed tomography (CT) of the head and magnetic resonance imaging (MRI) brain was normal without any evidence for acute ischemic changes on diffusion‐weighted imaging (DWI) (Figure 1A,B), and T2 signal changes on the fluid‐attenuated inversion recovery (FLAIR) sequence (Figure 1C). CT angiogram (CTA) of the head and neck revealed complete occlusion of the left subclavian artery distal to its origin and proximal to the origin of the left vertebral artery (Figure 1D). Metabolic, autoimmune, infectious, paraneoplastic, and neoplastic workup was negative (Appendix S1). The patient had mildly elevated phenytoin levels, but that was not causative of her symptoms. The patient's symptoms persisted for over 2 months without any improvement. She underwent left carotid artery to subclavian artery bypass with complete resolution of neurological symptoms.

**FIGURE 1:**
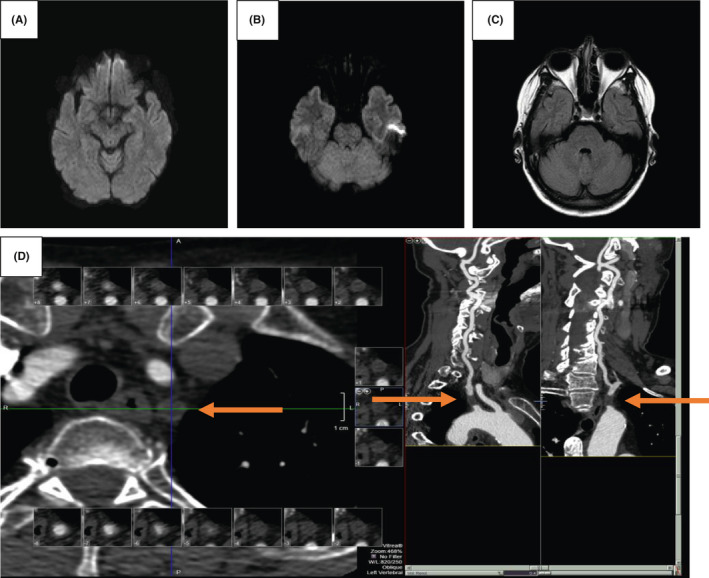
Brain MRI shows no diffusion restriction in the posterior circulation on DWI sequence (A and B), without associated T2 hyperintensity on fluid‐attenuated inversion recovery sequence (C). CTA neck shows complete occlusion of the left subclavian artery distal to its origin and proximal to the origin of the left vertebral artery (D)

## 1 CONCLUSION

We present an unusual case of subclavian steal syndrome presenting with chronic vertebrobasilar insufficiency that improved after surgical revascularization. Subclavian steal syndrome is a vascular disorder that consists of significant blood supply restriction with resultant insufficiency of the vertebrobasilar artery and the subclavian artery causing symptomatic insufficiency to the brain and upper extremity. It is important to recognize this condition in patients with subacute to chronic posterior circulation vascular insufficiency as early diagnosis and treatment can have good clinical outcomes.^1^

## CONFLICT OF INTEREST

The authors have declared no conflicts of interests.

## AUTHOR CONTRIBUTIONS

SP: contributed to major role in the acquisition of data, design, and conceptualized study, analyzed the data, and helped drafting the manuscript. AM: contributed to drafting/revising of the manuscript for content including medical writing for content, major role in acquisition of data, study concept for design, analysis or interpretation of data, and approval of the final draft.

## ETHICAL APPROVAL

Informed consent has been obtained for the publication of this clinical image. Henry Ford Hospital does not require IRB approval for the case report.

## Supporting information

App S1

## Data Availability

Information related to this article is available from the corresponding author upon request.
