# Supplementary material for: Chronic vertebrobasilar insufficiency in subclavian steal syndrome
Source: Clin Case Rep. 2021 Feb 4;9(3):1828–30. doi: 10.1002/ccr3.3891 (PMC7981698; doi:10.1002/ccr3.3891)
Supplement: Supplementary file 1 — App S1 [file CCR3-9-1828-s001.docx]

**Appendix S1**

**Supplementary table:**

Laboratory data:

|  | Ref. Range | Results |
| --- | --- | --- |
| ANA | Latest Ref Range: Negative | Positive |
| ANA PATTERN | Unknown | Speckled |
| ANA TITER 1 | Latest Ref Range: <1:80 Titer | 1:160 |
| C-ANCA  P-ANCA | Latest Ref Range: <1:20 Titer | <1:20 |
| GAD Antibodies | Latest Ref Range: <5 IU/mL | <5 |
| MAG AB (IgM) | Negative | Negative |
| SS A/Ro ab | Negative | Negative |
| SSA B/La ab | Negative | Negative |
| ENA | Negative | Negative |
| Phenytoin total | 10-20 ug/ml | 23.4 |
| Phenytoin free | 1-2 ug/ml | 2.4 |

|  | Ref. Range | Results |
| --- | --- | --- |
| Sodium | 135 - 145 mmol/L | 138 |
| Potassium | 3.5 - 5.0 mmol/L | 3.4 (L) |
| Chloride | 98 - 111 mmol/L | 104 |
| CO2 | 21 - 35 mmol/L | 26 |
| Anion Gap | 3 - 13 | 8 |
| Blood Urea Nitrogen | 10 - 25 mg/dL | 6 (L) |
| CREATININE | <1.16 mg/dL | 0.54 |
| Calcium | 8.6 - 10.4 mg/dL | 8.7 |

|  | Ref. Range | Results |
| --- | --- | --- |
| Corrected Calcium | 8.7 - 10.1 mg/dL | 9.5 |
| ALT/SGPT | <52 IU/L | 24 |
| AST/SGOT | <35 IU/L | 32 |
| Albumin | 3.2 - 4.6 g/dL | 4.3 |
| PROTEIN TOTAL | 6.0 - 8.3 g/dL | 6.5 |
| Bilirubin, Total | <1.2 mg/dL | 0.3 |
| Alkaline Phosphatase | 40 - 140 IU/L | 146 (H) |
| Globulin | 1.7 - 3.6 g/dL | 2.2 |
| A/G Ratio | 0.9 - 1.8 | 2.0 (H) |
| GFR African American | >60 ml/min/1.73m2 | 110 |
| GFR NonAfrican American | >60 ml/min/1.73m2 | 95 |
| Lactate | < 2.1 mmol/L | 0.5 |

**CSF results:**

|  | Ref. Range | Results |
| --- | --- | --- |
| Tube # | Unknown | 4 |
| Volume | Latest Units: mL | 2.0 |
| Color | Unknown | Colorless |
| Clarity | Unknown | Clear |
| Spun Appearance | Unknown | Colorless (A) |
| Albumin, CSF | Latest Ref Range: 10 - 31 mg/dL | 19.6 |
| Glucose, CSF | Latest Ref Range: 40 - 80 mg/dL | 53 |
| Lactic Acid, CSF | Latest Ref Range: 1.2 - 2.4 mmol/L | 1.4 |
| Protein, CSF | Latest Ref Range: 15 - 55 mg/dL | 28 |
| RBC | Latest Ref Range: 0 /cu mm | 12 (H) |
| WBC | Latest Ref Range: 0 - 5 /cu mm | 7 (H) |
| Neutrophils, CSF | Latest Ref Range: 0 - 6 % | 50 (H) |
| Basophils | Latest Ref Range: 0 % | 0 |
| Eosinophils | Latest Ref Range: 0 % | 0 |
| Lymphocytes | Latest Ref Range: 40 - 80 % | 25 (L) |
| Macrophages | Latest Ref Range: 0 % | 0 |
| Monocytes | Latest Ref Range: 15 - 45 % | 25 |
| Mononucleates | Latest Ref Range: 15 - 45 % | 0 (L) |
| IgG, CSF | Latest Ref Range: 0.5 - 6.1 mg/dL | 6.4 (H) |
| B. Burgdorferi Abs (EIA), CSF | Latest Ref Range: <=0.99 LIV | 0.01 |
| VDRL, CSF | Unknown | Rpt |
| VDRL,CSF | Unknown | NONREACTIVE |
| West Nile IgG Abs CSF | Unknown | <1.30 |
| West Nile IgM Abs CSF | Unknown | <0.90 |
| WEST NILE VIRUS, CSF | Unknown | Rpt |
| IgG, Serum | Latest Ref Range: 700 - 1,600 mg/dL | 696 (L) |
| Albumin | Latest Ref Range: 3,848 - 5,304 mg/dL | 3,383 (L) |
| IgG Index/CSF | Latest Ref Range: 0.3 - 0.7 Ratio | 1.6 (H) |
| CSF oligoclonal bands | Negative | Negative |

| CMV DNA, PCR | Not detected | Not detected |
| --- | --- | --- |
| EBV | Not detected | Not detected |
| HSV 1 DNA | Not detected | Not detected |
| HSV 2 DNA | Not detected | Not detected |
| HSV PCR | Not detected | Not detected |
| CSF culture gram stain | Negative | Negative |
| CSF AFB smear | Negative | Negative |
| VZV DNA | Negative | Negative |

| **Paraneoplastic Autoantibody Eval, S** **(Mayo clinic Panel)**  Interpretive Comments  The following antibody was identifed: Neuronal AChR,  Ganglionic (Alpha-3).  * This profile, in the proper clinical context, would  support neurological autoimmunity.  * The positive predictive value for an autoimmune    neurological diagnosis (diverse phenotypes) among  patients with an alpha-3 ganglionic AChR antibody value  of 0.03?0.09 nM is 46%.  * The influence of monoclonal and polyclonal gammopathies  is uncertain.  * A paraneoplastic basis should be considered, according  to age, sex, and other risk factors.  * The positive predictive value for a cancer diagnosis  (diverse types) among patients positive for alpha-3  ganglionic AChR antibody is 30%  approximately 24% are  historical neoplasms, and 6% are detected prospectively.  * References: McKeon A, Lennon VA, Lachance DH, Fealey RD,  Pittock SJ. (2009). The ganglionic acetylcholine receptor  autoantibody: oncological, neurological and serological  accompaniments. Arch Neurol 2009 66(6):735?741.   Anti-Neuronal Nuclear Ab, Type 1  ANNA-1, S         Negative        titer   <1:240  Reflex Added      None.       Anti-Neuronal Nuclear Ab, Type 2  ANNA-2, S         Negative        titer   <1:240   Anti-Neuronal Nuclear Ab, Type 3  ANNA-3, S         Negative        titer   <1:240   Anti-Glial Nuclear Ab, Type 1  AGNA-1, S         Negative        titer   <1:240   Purkinje Cell Cytoplasmic Ab Type 1  PCA-1, S          Negative        titer   <1:240   Purkinje Cell Cytoplasmic Ab Type 2  PCA-2, S          Negative        titer   <1:240   Purkinje Cell Cytoplasmic Ab Type Tr  PCA-Tr, S         Negative        titer   <1:240   Amphiphysin Ab, S Negative        titer   <1:240   CRMP-5-IgG, S     Negative        titer   <1:240   Striational (Striated   Muscle) Ab, S   Negative        titer   <1:120   P/Q-Type Calcium   Channel Ab      0.00            nmol/L  <=0.02     N-Type Calcium   Channel Ab      0.00            nmol/L  <=0.03   ACh Receptor (Muscle)   Binding Ab      0.00            nmol/L  <=0.02   AChR Ganglionic   Neuronal Ab, S  0.05     H      nmol/L  <=0.02   Neuronal (V-G) K+   Channel Ab, S   0.00            nmol/L  <=0.02   ADDITIONAL INFORMATION  This test was developed and its performance characteristics  determined by Mayo Clinic in a manner consistent with CLIA  requirements. This test has not been cleared or approved by  the U.S. Food and Drug Administration.   Test(s) performed at  Mayo Clinic Laboratories - Rochester Main Campus  200 First St SW Rochester, MN 55905    **CT Chest 8/22/2019- Initial CT for malignancy screening**  IMPRESSION:  1. Peripheral right upper lobe cystic lucency with adjacent focal  Ground glass. This could represent a bulla with adjacent scarring or  fibrosis, however cystic lung malignancy cannot be excluded. Compare  with outside CT chest examinations if available to document stability.  If outside CT examinations are not available, a follow-up low-dose  chest CT is recommended in 3 months to evaluate for stability or  resolution.  2. Unchanged proximal left subclavian artery occlusion. The vessel  reconstitutes proximal to the origin of the left vertebral artery  which is well opacified.  3. Similar 1 cm right thyroid nodule. Nonemergent thyroid ultrasound  evaluation is recommended. |
| --- |
| **Repeat CT chest 8/31/2020- for surveillance**  IMPRESSION  1.  Few punctate (1-2 mm) solid pulmonary nodules, and possible 13 mm ground glass nodule in the right upper lobe (versus scarring adjacent to a small bulla).  In this patient who is eligible for lung cancer screening, this would be considered Lung RADS category 2 (benign appearance).  Follow-up CT scan in 12 months is advised.  2.  Cholelithiasis. |
